# The combination of Biochanin A and SB590885 potentiates the inhibition of tumour progression in hepatocellular carcinoma

**DOI:** 10.1186/s12935-020-01463-w

**Published:** 2020-08-05

**Authors:** Yi Xiao, Qiang Gong, Wenhong Wang, Fang Liu, Qinghong Kong, Feng Pan, Xiaoke Zhang, Changyan Yu, Shanshan Hu, Fang Fan, Sanhua Li, Yun Liu

**Affiliations:** 1 Guizhou Provincial College-based Key Lab for Tumor Prevention and Treatment with Distinctive Medicines, Zunyi Medical University, No.6 West Xuefu Road, Xinpu District, Zunyi, 563000 China; 2 Department of Biochemistry and Molecular Biology, Zunyi Medical University, No.6 West Xuefu Road, Xinpu District, Zunyi, 563000 China; 3 Research Center for Medicine & Biology, Zunyi Medical University, Zunyi, 563000 China; 4 Good Clinical Practice Center, Affiliated Hospital of Zunyi Medical University, Zunyi, 563000 China

**Keywords:** Biochanin A, SB590885, Hepatocellular carcinoma, Combination therapeutic, ERK MAPK pathway

## Abstract

**Background:**

Hepatocellular carcinoma (HCC) is the most aggressive and frequently diagnosed malignancy of the liver. Despite aggressive therapy, life expectancy of many patients in these cases is extended by only a few months. Hepatocellular carcinoma (HCC) has a particularly poor prognosis and would greatly benefit from more effective therapies.

**Methods:**

The CCK-8 assay and colony formation assays were used to test the cell proliferation and viability. The effects of combination Biochanin A and SB590885 on apoptosis and cell cycle arrest of HCC cells were analysed by flow cytometry. The expression of ERK MAPK and PI3K/AKT/mTOR signalling as well as apoptosis and cell cycle-related proteins in HCC cells were tested by western blotting. The HCC cell xenograft model was established to test the tumor proliferation. Serum and plasma were tested for liver and kidney safety markers (ALP, ALT, AST, total bilirubin, creatinine, urea nitrogen) by using SpectraMax i3X.

**Results:**

The combination of natural product Biochanin A with the BRAF inhibitor SB590885 synergistically suppressed proliferation, and promoted cell cycle arrest and apoptosis in vitro. Furthermore, we demonstrated that the combination of Biochanin A and SB590885 led to increased impairment of proliferation and HCC tumour inhibition through disrupting of the ERK MAPK and the PI3K/AKT pathways in vitro. The volumes tumors and the weights of tumours were significantly reduced by the combination treatment compared to the control or single treatments in vivo. In addition, we found that there was no significant hepatorenal toxicity with the drug combination, as indicated by the hepatorenal toxicity test.

**Conclusion:**

The results identify an effective combination therapy for the most aggressive form of HCC and provide the possibility of therapeutic improvement for patients with advanced HCC.

## Background

Hepatocellular carcinoma (HCC) is identified as the fifth most common cause of cancer related mortality in worldwide and the prognosis for HCC remains very poor [1–6]. Sorafenib, a chemotherapeutic agent, is an approved drug available for liver cancer treatment. The reduced sensitivity of cancer cells and drug resistance are becoming more common, because sorafenib is used continuously. However, the efficacy of targeted therapies is limited due to drug resistance and acute cytotoxicity [7, 8]. Therefore, novel therapeutic strategies for HCC to address drug resistance and toxicity are urgently needed.

The development of drug resistance restricts the efficacy of single therapeutic agents [9]. Thus, new approaches that inhibit tumour growth have major clinical significance. Combinations of different therapeutic agents to inhibit several pathways could be a more effective strategy for suppressing cancer and could be more effective for treating cancer patients than single therapeutic agents. The natural iso–flavonoid (biochanin A) is found in red clover, chick peas, and several other plant sourcesis catego-rized as a phytoestrogen and has been demonstrated to exhibit various pharmacological properties [10, 11]. Biochanin A is known to exhibit an anticancer effect against various cancer types. Previous studies have shown that biochanin A inhibits endothelial cell functions such as cell viability, migration, and invasion through inhibition of the ERK/AKT/mTOR signalling pathway [10]. Recent studies have demonstrated that biochanin A treatment reduced cancer progression by inhibiting cancer cell proliferation, cellular signalling, invasion, and antioxidant systems [12–14] in colon cancer [15], prostate cancer [16], hepatoma [17] and human pharynx squamous carcinoma cells [18]. The possibility of MAPK pathway inhibition would have therapeutic benefits in patients with oncological diseases who have continous activation of this signalling pathway [19, 20]. RAF inhibitors generally exhibit greater response rates in clinical trials than MEK inhibitors which may be related to ERK activity suppression. SB590885 is a novel triarylimidazole that selectively inhibits RAF kinases with more potency towards the BRAF active conformation than the inactive conformation [19, 21]. Previous studies have shown that the combination of SB590885 and the AKT inhibitor ZSTK474 impacted the proliferation of papillary thyroid cancer cell lines by inhibiting the ERK MAPK and PI3K/AKT signalling pathways [19, 22]. However, whether the combination of biochanin A and SB590885 inhibits hepatocellular carcinoma (HCC) growth remains a blank box.

Therefore, to develop a therapeutic strategy that could provide an improvement in the treatment of advanced HCC without increased toxicity, we investigated the effects of combina-tions of biochanin A with the BRAF inhibitor SB590885 on anti-proliferative and survival pathways in HCC cells in vitro and in vivo.

## Materials and methods

### Cell culture

The HCC cell lines Bel-7402 and SK-Hep-1 were obtained from Shanghai Genechem Co. (Shanghai, China). The STR genotyping reports of the HCC cell lines Bel‑7402 and SK-Hep-1 are listed in Additional file 1: Tables S3 and S4. The cell lines were cultured in RPMI-1640 medium supplemented with 1% penicillin/streptomycin (Invitrogen, Carlsbad, CA, USA) and 10% fetal bovine serum (Gibco, Carlsbad, CA, USA) and were maintained at 37 °C in a humidified incubator containing 5% CO_2_.

### Cell viability assay

Biochanin A and SB590885 were purchased from Targetmol (Shanghai, China). Cell viability was tested using the CCK‑8 assay according to the manufacturer’s instructions. Cells (7 × 10^3^) were seeded into a 96‑well plate and cultured in regular growth medium containing 10% foetal bovine serum. After 24 h, the cells were exposed to serial dilutions of biochanin A (12.5 μM, 25 μM, 50 μM, 75 μM, and 100 μM), SB590885 (3 μM, 6 μM, 9 μM, 12 μM, and 15 μM). After the cells were incubated at 37 °C for 48 h, the medium was removed and 100 μl of RPMI‑1640 and 10 μl of CCK‑8 reagent were added. The cells were incubated for 2 h at 37 °C. Finally, the absorbance of each well was measured at 450 nm using SpectraMaxi3X (Molecular Devices, Silicon Valley, CA, USA). All cell viability assays were performed at least three times. The combination index (CI), which was calculated by the Chou–Talalay equation, indicates synergistic effects at CI < 1, additive effects at CI = 1, and antagonism at CI > 1 [23].

### Colony formation assay

The HCC cell lines Bel‑7402 and SK-Hep-1 were incubated in a six-well plate with 75 μM biochanin A and 12 μM SB590885 for 10 days as previously described [22, 24]. The colonies were stained with crystal violet. The visible colonies were photographed by digital single lens reflex (Nikon D5600) and counted using ImageJ software (National Institutes of Health, Bethesda, MD, USA).

### Cell cycle and apoptosis analysis

The HCC cell lines Bel‑7402 and SK-Hep-1 were incubated in complete cell culture medium with 75 μM biochanin A and 12 μM SB590885 for 48 h. The cells were collected in cold PBS and then incubated with150 μl of RNase A (10 μg/ml) for 30 min at 37 °C in the dark, stained with 400 μl propidium iodide (PI) (50 μg/ml) and placed at 4 °C in the dark for 30 min [24, 25]. The stained cells were analysed using a flow cytometer (BD Bioscience, CA, US) [25]. For apoptosis analyses, high-affinity Annexin-V (AV) and PI (BD Biosciences, CA, USA) were used as previously described [26].

### Western blot analysis

SK-Hep1 and Bel-7402 cells were plated into 6-well plates for 24 h, and then treated with 75 µM biochaninA,12 µM SB590885 or the combination for 48 h. Total protein from the cells was extracted using RIPA buffer with a proteinase inhibitor (Beyotime, China). Protein samples were subjected to SDS–PAGE and then transferred to PVDF membranes (Millipore, US). The membranes were blocked with 5% BSA for 1 h, and incubated with primary antibodies such as Bcl-2 (1:1000), Bax (1:1000), cleaved PARP (1:1000), cleaved caspase-9 (1:1000), P21 (1:1000), P27 (1:1000), CyclinD1 (1:1000), p-MEK (1:1000), MEK (1:1000), p-ERK (1:1000), ERK (1:1000), AKT (1:1000), p-AKT (1:1000), P70S6K (1:1000), p-P70S6K (1:1000), S6 (1:1000), p-S6 (1:1000), β-actin (1:1000) (Cell Signaling Technology, Inc. (Danvers, MA, USA)) and GAPDH (1:1000)(Qianchen Biotech, Shanghai) at 4 °C overnight. The membranes were washed three times and incubated with the secondary anti-rabbit or anti-mouse antibody for 1.5 h at room temperature. Protein bands were visualized by Bio-Rad ChemiDoc Imaging System.

### Xenograft model analysis and groups of treatment

The HCC cell xenograft model was established as previously described [27]. Briefly, 5 × 10^6^ Bel-7402 cells were injected subcutaneously into the right flank near the hind leg of each nude mouse until the tumor volume was ~ 100 mm^3^. Biochanin A was dissolved in dimethyl sulfoxide (DMSO) at stock concentration of 100 mM, and stored in the dark at − 20 °C. SB590885 was dissolved in a 10 mM stock solution in DMSO and stored in the dark at − 20 °C. Then, we treated male athymic nude mice bearing palpable Bel-7402 xenografts tumours (~ 100 mm^3^) with control (vehicle-treated mice) 50 μl 0.5% dimethyl sulfoxide (DMSO), biochanin A (25 mg/kg/day), SB590885 (7.5 mg/kg/day) and the combination by intraperitoneal injection for 5 weeks, 7 times/week. The tumour volume was measured every week and was calculated by the following formula: volume = 1/2 (length × width^2^). After 5 weeks, the mice were euthanized and the tumours were isolated.

### Immunohistochemistry

Tumor xenografts were formalin-fixed, paraffin-embedded, and sectioned in preadherent slides. The slides were subjected to the indicated primary antibody against PCNA (1:200) (Cell Signalling Technology, Inc. (Danvers, MA, USA)) overnight. The sections were incubated with secondary antibody and then developed with 3,3′-diaminobenzidine chromogen according to the protocol. All samples were visualized, and images were captured using a DM2500 fluorescence microscope (Danaher, Wetzlar, Germany). Image‑Pro Plus 4.5 software was used to analyse the staining data.

### Liver and kidney toxicity analysis

Serum and plasma were tested for liver and kidney safety markers [28] (ALP, ALT, AST, total bilirubin, creatinine, and urea nitrogen; Additional file 1: Table S2) by using a SpectraMaxi3X(Molecular Devices, Silicon Valley, CA, USA).

### Statistical analysis

Statistical analyses were performed using SPSS 17.0 software (IBM, Armonk,New York, USA). Graphs were generated with GraphPad Prism 7.0 software (GraphPad, San Diego, CA, USA). All experiments were performed three independent times. Statistical significance was determined by Student’s t-test, and a two-sided *P*-value< 0.05 was considered statistically significant.

## Results

### Biochanin A synergizes with SB590885 to inhibit the viability and clonogenic potential of hepatocellular carcinoma cells

**Fig. 1:**
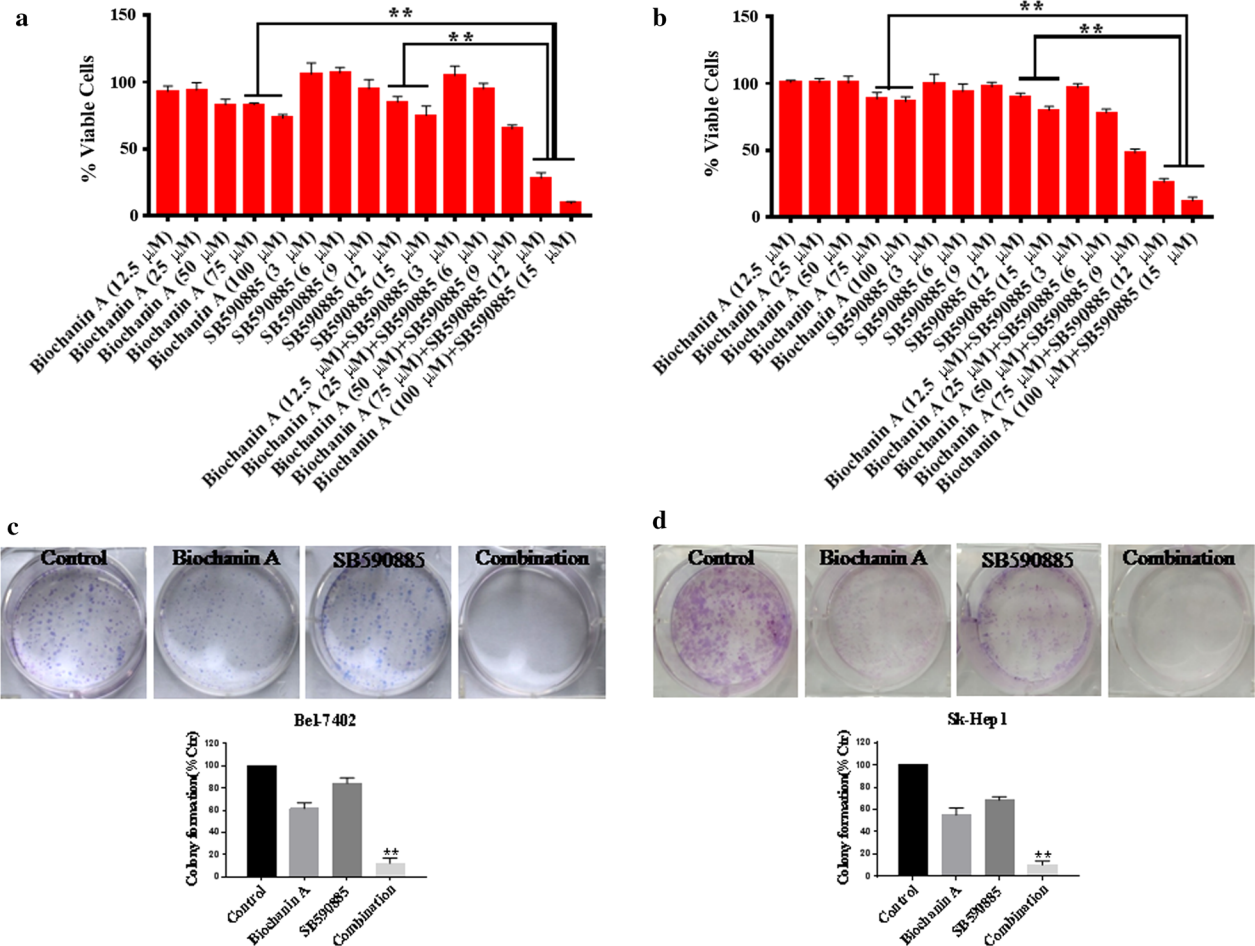
Anti-proliferative effects of combination treatments in HCC cells. After treatment with biochanin A (12.5 μM, 25 μM, 50 μM, 75 μM, and 100 μM) and SB590885 (3 μM, 6 μM, 9 μM, 12 μM, and 15 μM) alone or in combination for 48 h, the CCK‑8 assay was used to determine cell viability in the **a** Bel-7402 cell line and **b** SK-Hep1 cell line. The results are the mean ± SD of three independent experiments performed in triplicate. **P < 0.001 versus single treatments (one-way ANOVA followed by a Student–Newman–Keuls test). The combination of 75 μM biochanin A and 12 μM SB590885 significantly inhibited colony formation in HCC cell lines **c** Bel-7402 and **d** SK-Hep1. The results are the mean ± SD of three independent experiments performed in triplicate. **P < 0.001 versus single treatments (one-way ANOVA followed by a Student–Newman–Keuls test)

To determine the effect of the combination treatment on hepatocellular carcinoma, we investigated the effects on cellular morphology using microscopic inspection and the viability of Bel-7402 and SK-Hep-1 cells using the CCK-8 assay. We found that single treatment with biochanin A and SB590885 for 48 h suppressed the viability of Bel-7402 and SK-Hep-1 cells. The combination treatment significantly decreased the cell viability compared with that of the single treatment group (Fig. 1a, b, Additional file 1: Table S1). Moreover, the combination treatment also changed the cellular morphology and induced the cell death (Additional file 2: Figure S1). Furthermore, we performed the clonogenic assay, Over a 10 day treatment period, although the biochanin A and SB590885 single treated groups exhibited suppressed single cell colony progression, colony formation was almost eliminated in the combination group (Fig. 1c, d). Collectively, these results indicate that the combination of biochanin A and SB590885 synergistically inhibited the growth of hepatocellular carcinoma cells and induced the cell death.

### Combined treatment with biochanin A and SB590885 induced cell apoptosis in hepatocellular carcinoma cells

**Fig. 2:**
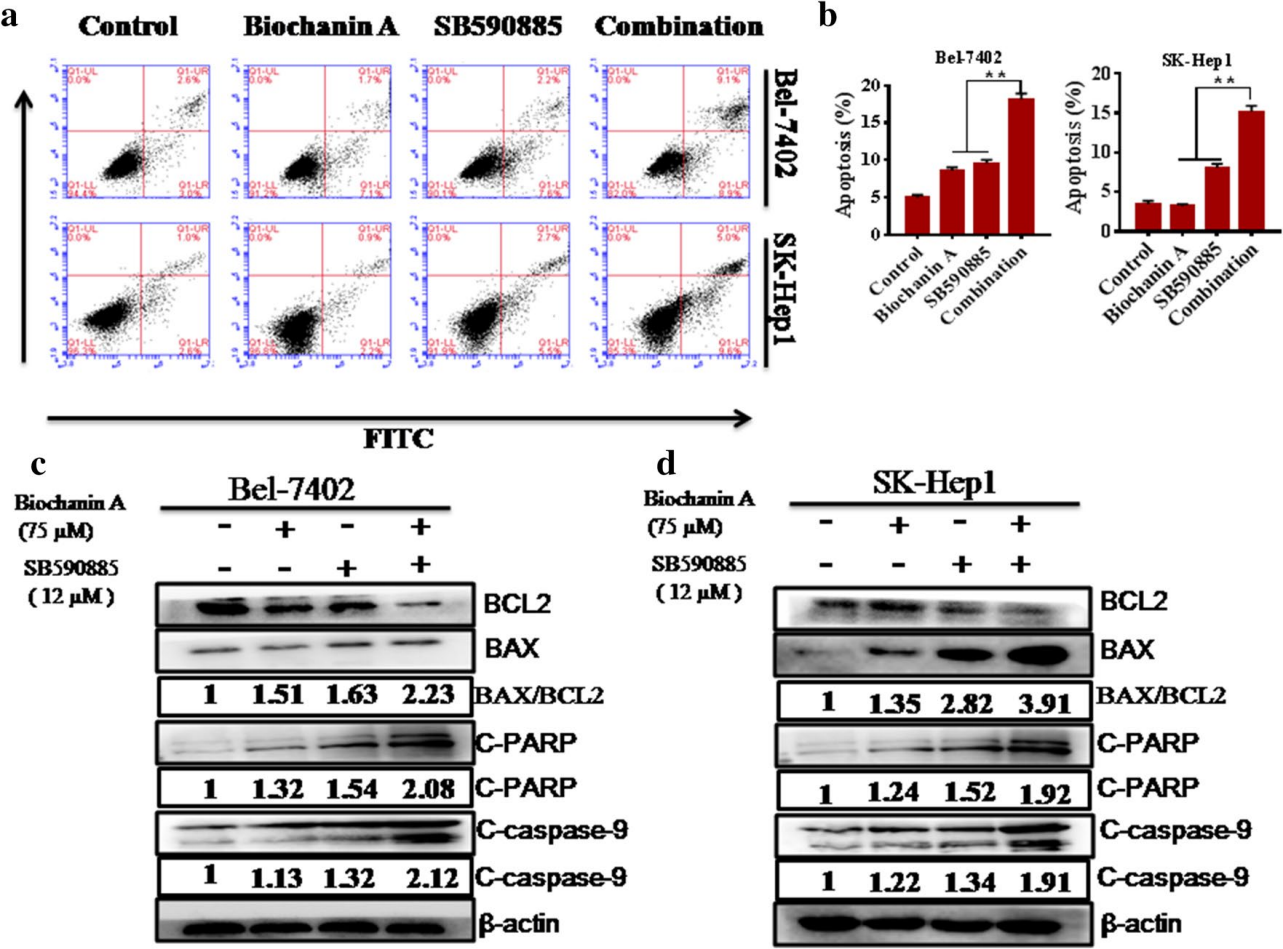
Effect of the combination treatment on HCC cell apoptosis. Synergistic activity of the combination treatment of biochanin A and SB590885 on apoptosis after 48 h of exposure in the HCC cell lines **a** Bel-7402 and **b** SK-Hep1 cell line. The results are the mean ± SD of three independent experiments performed in triplicate. **P < 0.001 versus single treatments (one-way ANOVA followed by a Student–Newman–Keuls test). BCL2, BAX, cleaved caspase-9 and cleaved PARP expression was measured in the **c** Bel-7402 cell line **d**, and SK-Hep-1 cell line using western blot analysis

To test whether the combination of biochanin A and SB590885 promoted apoptosis more effectively than the single treatment, we performed an apoptosis assay with FITC/PI staining and flow cytometry. Compared with each of the single agent treatments, combined treatment with biochanin A and SB590885 significantly increased the apoptotic proportion in Bel-7402 and SK-Hep-1 cells (Fig. 2a, b). Furthermore, to confirm these results, we evaluated the expression of BAX, cleaved caspase-3 and cleaved PARP in the combination treated group compared to the single treated group using western blot analysis (Fig. 2c, d). The ratio of BAX to BCL2 is an important indicator of apoptosis. Our results show that the ratio of BAX to BCL2 is increased in combination treated group compared to single treated group (Fig. 2c, d). These results suggest that biochanin A synergized with SB590885 and promoted apoptosis in HCC cells.

### Combined treatment with biochanin A and SB590885 induced cell cycle arrest in hepatocellular carcinoma cells

**Fig. 3:**
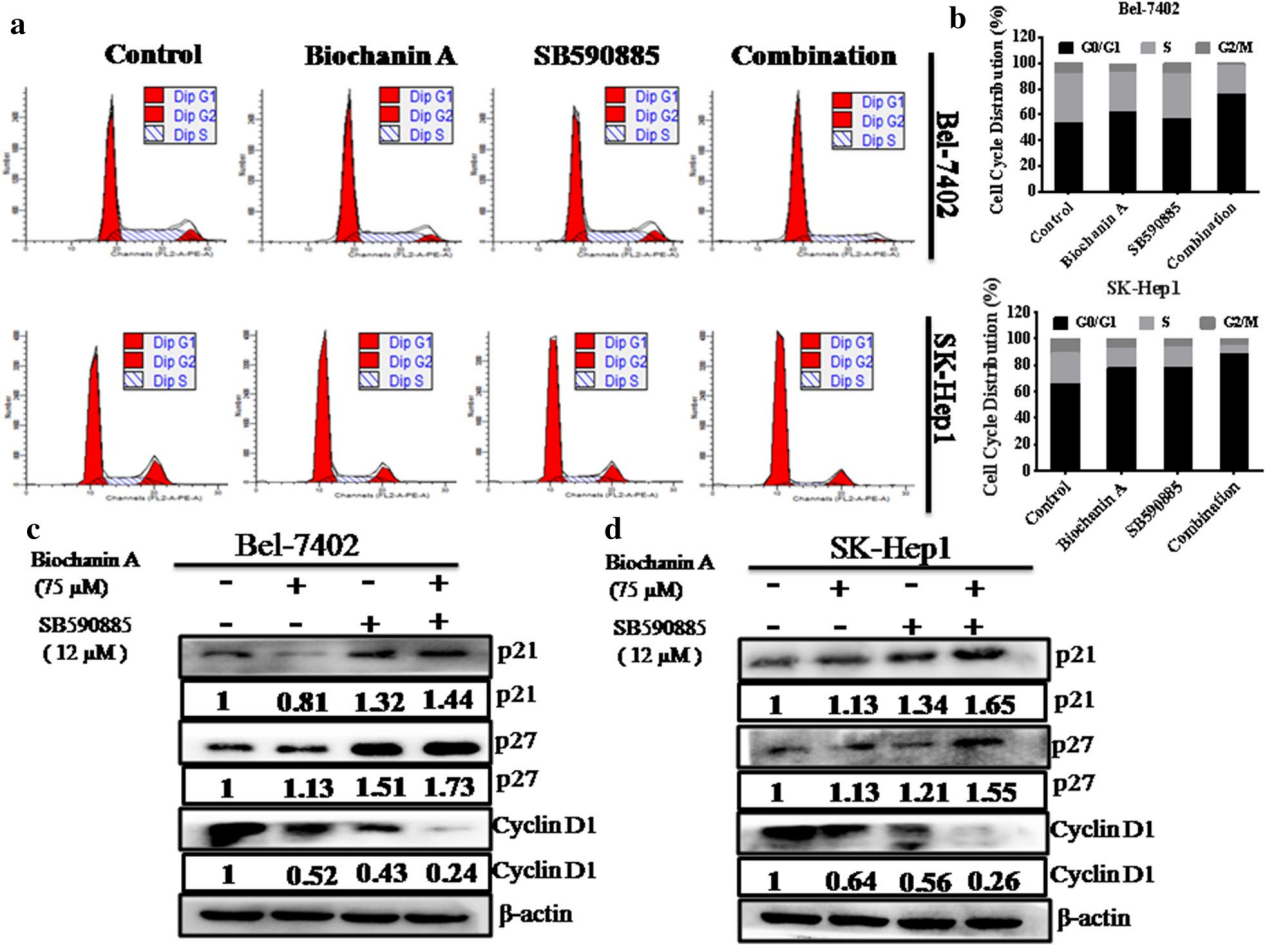
Effect of the combination treatment on HCC cell cycle arrest. Synergistic activity of the combination treatment of biochanin A and SB590885 on cell cycle arrest after 48 h of exposure in HCC cell lines **a** Bel-7402 and **b** SK-Hep1. The results are the mean ± SD of three independent experiments performed in triplicate. **P < 0.001 versus single treatments (one-way ANOVA followed by a Student–Newman–Keuls test). The expression of p21, p27, and cyclinD1 in the **c** Bel-7402 cell line and **d** SK-Hep1 cell line were tested using western blot analysis

We performed cell cycle progression analysis after drug treatment. Flow cytometry analyses demonstrated that combination treatment promoted cell cycle arrest in the G0/G1 phase (Fig. 3a, b). To confirm these results, we performed western blot analysis and found that the expression of p21 and p27, which are cyclin dependent kinases, was markedly enhanced in the combination treatment group compared to the single treatment group (Fig. 3c, d). In contrast, the level of cyclin D1, a key regulators of G0/G1 to S phase transition, was further reduced in the combination treatment group compared to single treatment group (Fig. 3c, d). These results suggest that the combination of biochanin A and SB590885 promoted cell cycle arrest in HCC cells.

### Combined treatment with biochanin A and SB590885 regulates the ERK MAPK pathway in hepatocellular carcinoma cells

**Fig. 4:**
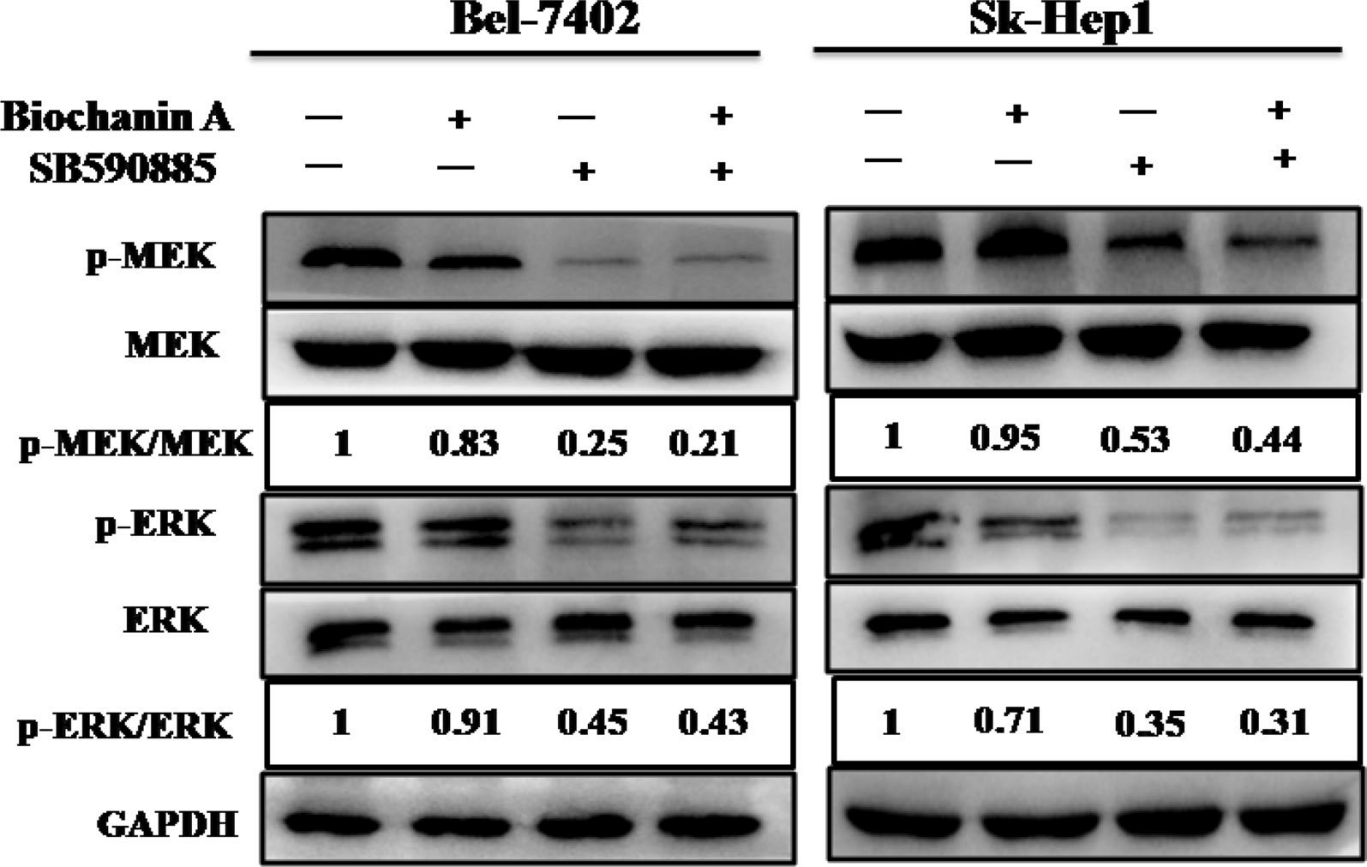
Effect of the combination treatment on the ERK MAPK pathway in HCCcell lines. The p-ERK, total-ERK, p-MEK and total-MEK levels in the Bel-7402 cell line and SK-Hep1 cell line were measured using western blot analysis. These results are performed with three independent experiments in triplicate

To determine the underlying molecular mechanism, we investigated the effects of biochanin A and SB590885 on the ERK MAPK pathway in HCC cells. We found that combination treatment with biochanin A and SB590885 significantly suppressed the phosphorylation levels of MEK and ERK in Bel-7402 and SK-Hep-1 cells (Fig. 4). These results suggest that the combination of biochanin A and SB590885 suppressed the growth of HCC cells via inhibition of the ERK MAPK signalling pathway.

### Combined treatment with biochanin A and SB590885 regulates the PI3K/AKT/mTOR pathway in hepatocellular carcinoma cells

**Fig. 5:**
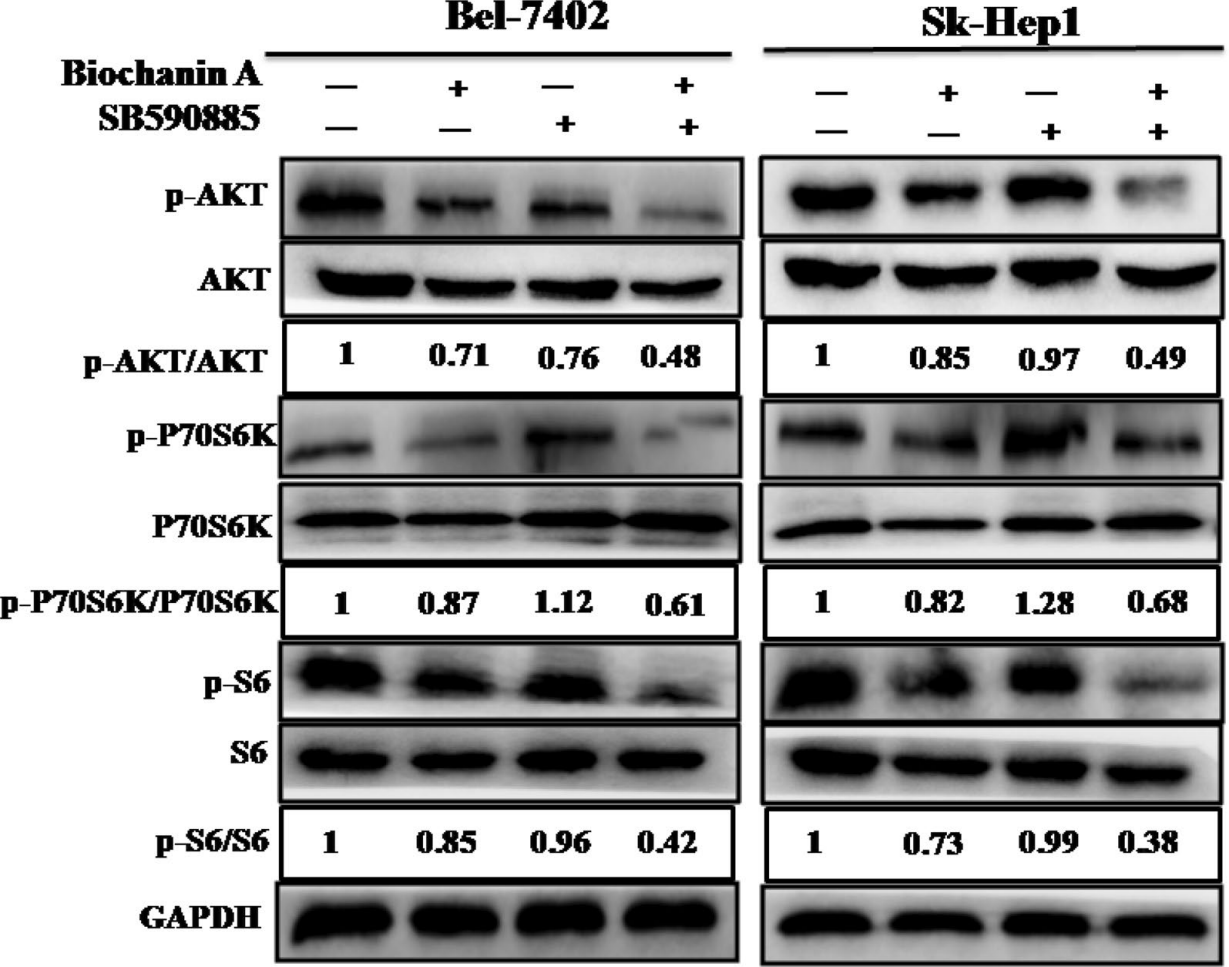
Effect of combination treatment on the PI3K/AKT/mTOR pathway in HCCcell lines. The p-AKT, total-AKT, p-P70S6K, total-P70S6K, p-S6 and total-S6 in the Bel-7402 cell line and SK-Hep1 cell line were measured using western blot analysis. These are the results of three independent experiments performed in triplicate

Single-agent treatment inhibited the ERK MAPK pathway, often resulting in reversing activation of the PI3K/AKT/mTOR pathway. Thus we investigated the effects of biochanin A and SB590885 on the PI3K/AKT/mTOR pathways in HCC cells. We found that the combination treatment of biochanin A and SB590885 significantly suppressed the phosphorylation levels of AKT, P70S6K and S6 in Bel-7402 and SK-Hep-1 cells (Fig. 5). These results suggest that the combination of biochanin A and SB590885 suppressed the growth of hepatocellular carcinoma cells via inhibition of the PI3K/AKT/mTOR signalling pathway.

### Combined treatment with biochanin A and SB590885 efficiently reduces HCC growth in an HCC xenograft model

**Fig. 6:**
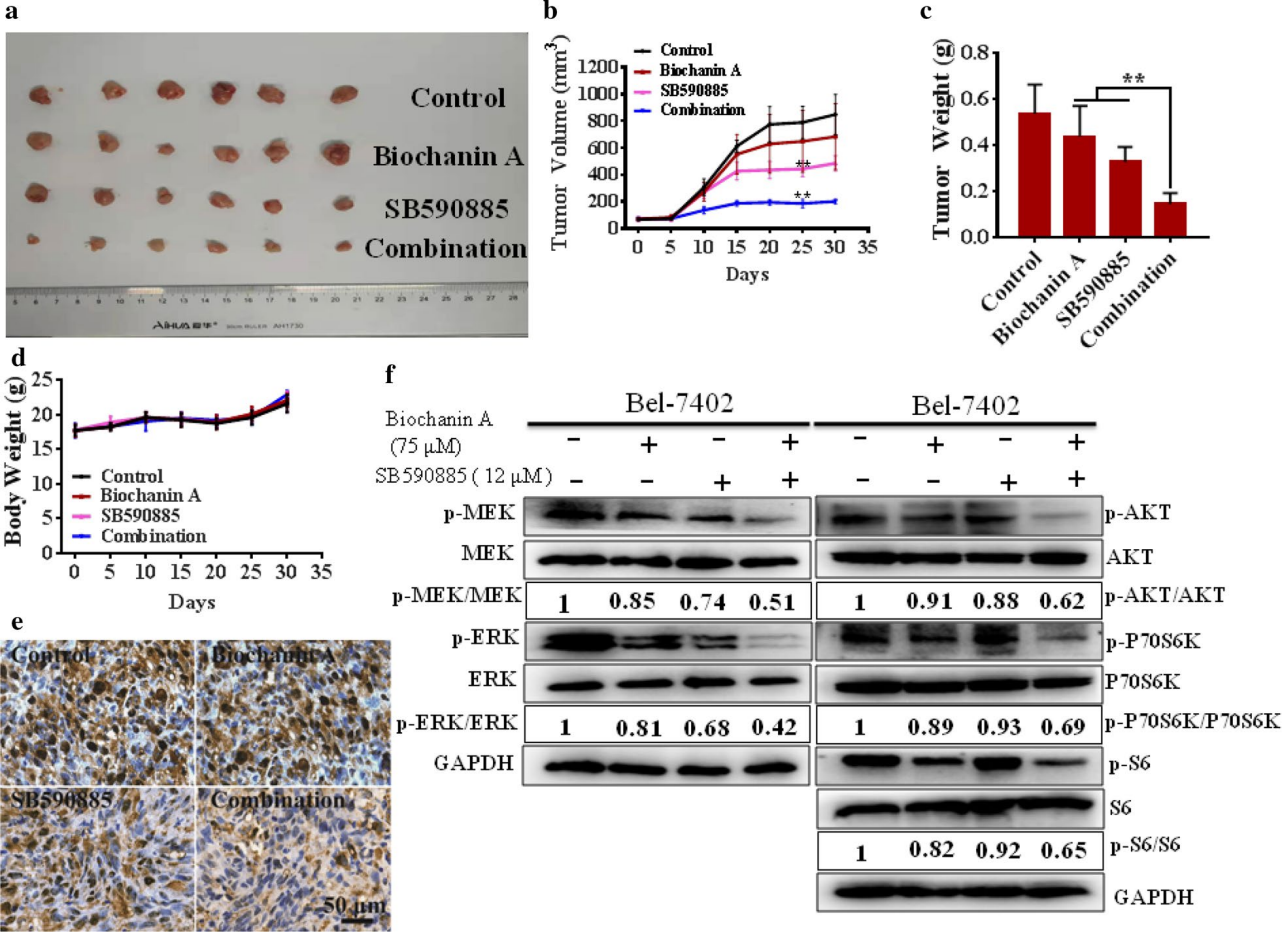
Anti-proliferative effects of the combination treatments in vivo. HCC xenograft model with treated with control (vehicle-treated mice), biochanin A (25 mg/kg/day), SB590885 (7.5 mg/kg/day) and the combination for 5 weeks. The size (**a**), volume (**b**) and weight of the tumours (**c**) were significantly reduced by the combination treatment compared to those of the control or single treatments. No significant differences in body weight were observed at the end of the treatment period (**d**). Immunohistochemical analyses of the xenograft tumours revealed that the biochanin A and SB590885 combination effectively inhibited the expression of PCNA, a marker of tumour proliferation (**e**). Western blot analysis of the xenograft tumours revealed that the combination of biochanin A and SB590885 inhibited activation of the ERK MAPK and PI3K/AKT/mTOR signalling pathways in the in vivo xenograft model (**f**). The results are the mean ± SD of three independent experiments performed in triplicate. **P < 0.001 versus single treatments (one-way ANOVA followed by a Student–Newman–Keuls test)

To test the effect of the combination treatment in vivo, we treated male athymic nude mice bearing palpable Bel-7402 xenografts tumours (~ 100 mm^3^) with control (vehicle-treated mice), biochanin A (25 mg/kg/day), SB590885 (7.5 mg/kg/day) and a combination of biochanin A and SB590885 for 5 weeks. The size, the volume and the weight of tumours were significantly reduced by the combination treatment compared to those of the control or single treatments (Fig. 6a–c). Furthermore, to characterize the safety of the combination of biochanin A and SB590885 in HCC xenograft model, nude mice bearing palpable Bel-7402 xenografts tumours were treated for 5 weeks with biochanin A, SB590885, and the combination of both or were untreated in the control group. No significant differences in body weight were observed at the end of the treatment (Fig. 6d, Additional file 1: Table S2). Immunohistochemical analyses of the xenograft tumours revealed that the biochanin A and SB590885 combination effectively inhibited the expression of PCNA, a marker of tumour proliferation (Fig. 6e). Western blot analysis of the xenograft tumours revealed that the combination of biochanin A and SB590885 inhibited activation of the ERK MAPK and PI3K/AKT/mTOR signalling pathways in the in vivo xenograft model (Fig. 6f). In addition, blood sample analysis indicated that none of the treatments affected kidney or liver functions. There was no significant difference in the levels of transaminases, alkaline phosphatase (ALP), total bilirubin, creatinine and urea nitrogen among all groups (Additional file 1: Table S2). Overall, these results indicate that the combination treatment of biochanin A and SB590885 inhibited tumour growth in the HCC xenograft model and did not influence kidney and liver functions.

## Discussion

The heterogeneity of HCC is characterized by both heritability [29] and phenotype/morphology [30]. Tumours are characterized by complex patterns such as location and timing. This complex and multivariable tumour network constantly responds to and affects the liver environment,which is the main reason for the limited success of different targeted single-therapy trials for HCC [31]. Therefore, combining multiple anticancer drugs seems to be a reasonable way to prevent tumour resistance. In this study, we provide evidence that combined treatments play a crucial role in the chemoprevention of HCC.

Chemotherapy is an important treatment option for various clinical factors. Among the chemotherapeutic agents, sorafenib is administered and is significant in the treatment of HCC. However, sorafenib only improves median overall survival by ~ 3 months [32–34]. Single–agent treatments are usually partial and relatively transient, and toxicity to normal tissues is one of the major obstacles to successful cancer chemotherapy [7, 35]. Several studies have shown that natural products play an important role in the inhibition and treatment of cancers [36, 37]. Thus, there has been increasing focus on the application of combined treatments using natural products for HCC. For example, a previous study showed that biochanin A combined with ginsenoside Rh2 exhibited synergistic effects against MDA-MB-231 and MCF-7 cell proliferation [37]. Biochanin A, an isoflavone, has been shown to exert anticancer effects against various cancers. SB590885 is a serine/threonine-protein kinase B-Raf (BRAF) inhibitor. Our results indicated that the combination of biochanin A and (the BRAF) inhibitor SB590885 significantly inhibited the proliferation and viability of HCC in vitro and in vivo. Apoptosis is a tightly regulated signalling process that involves the coordination of anti-apoptotic and pro-apoptotic proteins [38]. In this study, our results demonstrated that biochanin A combined with SB590885 induced apoptosis in HCC cells more markedly than either biochanin A or SB590885 alone. Intriguingly, a recent study showed that biochanin A induced S phase arrest in lung cancer cells [39]. However, we found that the combination of biochanin A and SB590885 induced apoptosis, along with G0/G1 phase cell cycle arrest in HCC cells. These results revealed that drugs acting on different cells might cause cell cycle arrest at different phases.

Several studies have suggested that HCC cell activation by different factors is known to increase both Ras/Raf/ERK MAPK and PI3K/AKT/mTOR signalling [1, 40]. Sorafenib, the drug available for the treatment of patients with advanced HCC, inhibits the Ras/Raf/MAPK pathway [32], but does not directly inhibit the PI3K/AKT/mTOR pathway, which also plays an important role in HCC proliferation. The PI3K pathway is known to be activated in 30% to 50% of HCC cases [41]. Somatic mutation of PIK3CA, enhancement of Akt expression and phosphorylation ribosomal protein S6, and a decrease in PTEN expression have been reported in HCC [42–45]. These studies suggest that combined targeting of the PI3K/AKT/mTOR and Ras/Raf/MAPK pathways might provide benefits in the treatment of HCC. Previous studies have shown that biochanin A inhibited endothelial cell functions such as cell viability, migration, and invasion through inhibition of the ERK/AKT/mTOR signalling pathway [10]. SB590885 is a serine/threonine-protein kinase B-Raf (BRAF)inhibitor. Previous studies have shown that the combination of SB590885 and the AKT inhibitor ZSTK474 impacted the proliferation of papillary thyroid cancer cell lines via inhibition of the ERK MAPK and PI3K/AKT signalling pathways [19, 22]. Our results indicated that the combination of biochanin A and SB590885 suppressed the growth of hepatocellular carcinoma cells via inhibition of the ERK MAPK and PI3K/AKT/mTOR signalling pathways. Furthermore, a hepatorenal toxicity test showed that the combination biochanin A and SB590885 did not induce any significant hepatorenal toxicity. The safety and efficacy of this combination strategy provides the possibility of improving of therapeutic outcomes for advanced HCC patients.

## Conclusion

In conclusion, our results show that combination of the natural product Biochanin A with the BRAF inhibitor SB590885 synergistically suppressed proliferation, and promoted cell cycle arrest and apoptosis in HCC cells. The combination of biochanin A and SB590885 inhibited the HCC cells proliferation through ERK MAPK and PI3K/AKT pathways. We found that there was no significant hepatorenal toxicity with the drug combination, as indicated by the hepatorenal toxicity test. These results indicate that combination Biochanin A and SB590885 is a potential treatment for HCC.

## Supplementary information

**Additional file 1: Table S1.** Synergistic indexes of combination treatment with Biochanin A and SB590885 in Bel-7402 and Sk-Hep1 hepatocellular carcinoma cell lines. **Table S2.** Clinical and biological analyses. **Table S3.** The STR test results of SK-Hep-1 cells. **Table S4.** The STR test results of Bel‑7402 cells.

**Additional file 2.** Representative photomicrographs of Bel-7402 and SK-Hep1 cells after 48 h of combination treatment biochanin A and SB590885.

## Data Availability

The datasets used and/or analyzed during the current study are available from the corresponding author on reasonable request.
