# Supplementary material for: The combination of Biochanin A and SB590885 potentiates the inhibition of tumour progression in hepatocellular carcinoma
Source: Cancer Cell Int. 2020 Aug 5;20:371. doi: 10.1186/s12935-020-01463-w (PMC7405455; doi:10.1186/s12935-020-01463-w)
Supplement: Supplementary file 1 — Additional file 1: Table S1. Synergistic indexes of combination treatment with Biochanin A and SB590885 in Bel-7402 and Sk-Hep1 hepatocellular carcinoma cell lines. Table S2. Clinical and biological analyses. Table S3. The STR test results of SK-Hep-1 cells. Table S4. The STR test results of Bel‑7402 cells. [file 12935_2020_1463_MOESM1_ESM.docx]

**Table S1** Synergistic indexes of combination treatment with Biochanin A and SB590885 in Bel-7402 and Sk-Hep1 hepatocellular carcinoma cell lines.

|  | Bel-7402 | |  | |  | Sk-Hep1 |  |
| --- | --- | --- | --- | --- | --- | --- | --- |
| Biochanin A  (μM) | | SB590885  (μM) | | CI | BiochaninA (μM) | SB590885  (μM) | CI |
| 12.5 | 3 | | 8.14 | | 12.5 | 3 | 0.65 |
| 25 | 6 | | 2.31 | | 25 | 6 | 0.48 |
| 50 | 9 | | 0.61 | | 50 | 9 | 0.35 |
| 75 | 12 | | 0.31 | | 75 | 12 | 0.27 |
| 100 | 15 | | 0.19 | | 100 | 15 | 0.22 |

**Table S2** Clinical and biological analyses.

|  | Control  (n=5) | BiochaninA (n=5) | SB590885  (n=5) | Combination  (n=5) | ANOVA  p-value |
| --- | --- | --- | --- | --- | --- |
| Body Weight  (g) | 18.2±1.2 | 17.9±1.4 | 19.2±1.1 | 18.5±1.6 | *ns* |
| ALT (U/L) | 20.4±2.3 | 24.2±2.6 | 18.5±1.8 | 21.3±2.4 | *ns* |
| AST (U/L) | 20.1±1.4 | 21.1±2.1 | 16.6±2.7 | 18.6±2.3 | *ns* |
| Total Bilirubin (μmol/L) | 15.7±2.2 | 14.2±2.6 | 16.4±2.5 | 12.9±2.8 | *ns* |
| ALP(U/L) | 156.8±6.2 | 154.8±5.9 | 189.2±7.3 | 144.7±5.4 | *ns* |
| Creatinine  (μmol/L) | 9.1±2.6 | 7.1±2.4 | 6.4±2.7 | 9.8±2.8 | *ns* |
| Urea Nitrogen (mmol/L) | 3.3±0.4 | 3.5±1.1 | 4.1±1.3 | 3.6±0.9 | *ns* |

Abbreviations: AST, aspartate aminotransferase; ALT, alanine aminotransferase; ALP, alkaline phosphatase. Values are means ± SE. No significant difference (*ns*) compared to control.

**Table S3** The STR test results of SK-Hep-1cells.

| Number | STR loci | Theoretical typing | Theoretical size | Actual size | Actual typing | Whether conform |
| --- | --- | --- | --- | --- | --- | --- |
| 1 | Amelogenin | X | 212 | 210.96, 211.89 | X | Yes |
| 2 | CSF1PO | 11, 12 | 311, 315 | 309.62, 313.83 | 11, 12 | Yes |
| 3 | D13S317 | 8, 12 | 169, 185 | 173.25, 189.43 | 9, 13 | No |
| 4 | D16S539 | 12 | 161 | 158.98 | 12 | Yes |
| 5 | D5S818 | 10, 13 | 145, 157 | 142.01, 155.07 | 10, 13 | Yes |
| 6 | TPOX | 9 | 236 | 234.2 | 9 | Yes |
| 7 | D7S820 | 8, 11 | 206, 218 | 204.39, 216.31 | 8, 11 | Yes |
| 8 | D21S11 | 29, 31 | 222, 230 | 222.32, 230.27 | 29, 31 | Yes |
| 9 | TH01 | 7, 9 | 187, 195 | 187.28, 194.95 | 7, 9 | Yes |
| 10 | vWA | 14, 17 | 139, 151 | 137.53, 151.07 | 14, 17 | Yes |

None of the 10 loci of the cell showed the phenomenon of third-class gene, indicating no cross contamination with other cells. The DNA typing degree of this strain was ≥80% in the ATCC cell bank (National Laboratory Cell Resource sharing Platform cell bank), and the cells should be SK-HEP-1 cells.

**Table S4** The STR test results of Bel‑7402cells.

| Number | STR loci | Theoretical typing | Theoretical size | Actual size | Actual typing | Whether conform |
| --- | --- | --- | --- | --- | --- | --- |
| 1 | Amelogenin | X | 212 | 213.03 | X | Yes |
| 2 | CSF1PO | 10 | 307 | 305.17, 306.35 | 10 | Yes |
| 3 | D13S317 | 13.3 | 192 | 199.71 | 15 | No |
| 4 | D16S539 | 9, 10 | 149, 153 | 146.78, 151.17 | 9, 10 | Yes |
| 5 | D5S818 | 12 | 153 | 151.27 | 12 | Yes |
| 6 | TPOX | 12 | 248 | 248.69 | 12 | Yes |
| 7 | D7S820 | 12 | 222 | 222.01 | 12 | Yes |
| 8 | D21S11 | 27, 28 | 214, 218 | 215.88, 220.27 | 27, 28 | Yes |
| 9 | TH01 | 7 | 187 | 187.69 | 7 | Yes |
| 10 | vWA | 16, 18 | 147, 155 | 147.18, 155.92 | 16, 18 | Yes |

None of the 10 loci of the cell showed the phenomenon of third-class gene, indicating no cross contamination with other cells. The DNA typing degree of this strain was ≥80% in the ATCC cell bank (National Laboratory Cell Resource sharing Platform cell bank), and the cells should be Bel‑7402 cells.
