# Supplementary figures and images for: The combination of Biochanin A and SB590885 potentiates the inhibition of tumour progression in hepatocellular carcinoma
Source: Cancer Cell Int. 2020 Aug 5;20:371. doi: 10.1186/s12935-020-01463-w (PMC7405455; doi:10.1186/s12935-020-01463-w)

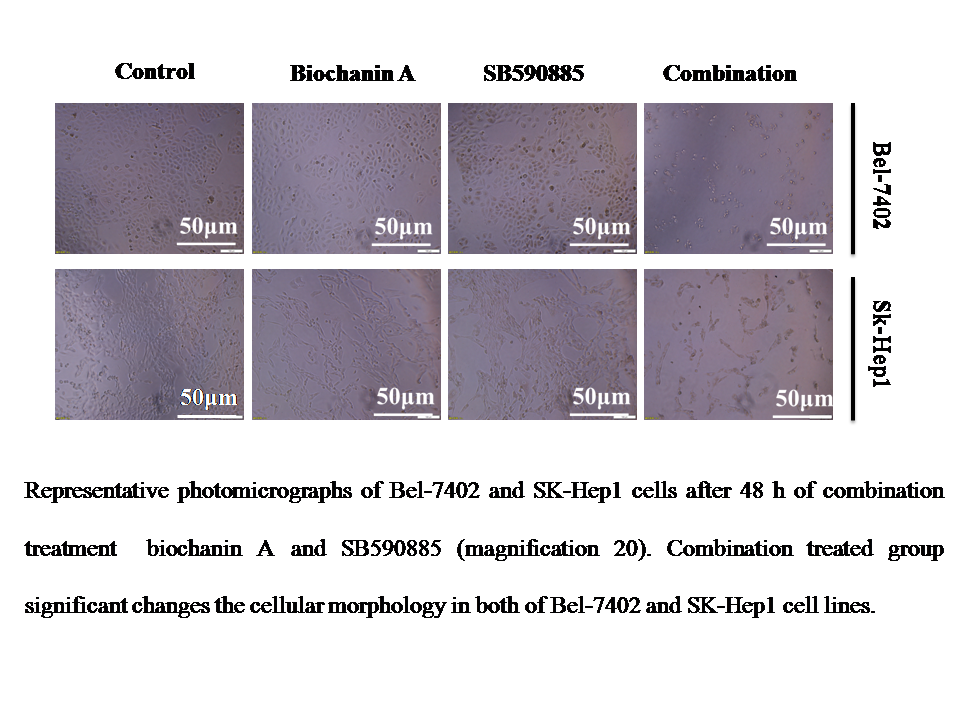

Supplement: Supplementary file 2 — Additional file 2. Representative photomicrographs of Bel-7402 and SK-Hep1 cells after 48 h of combination treatment biochanin A and SB590885. [file 12935_2020_1463_MOESM2_ESM.tif]
